# A lightweight detection algorithm of PCB surface defects based on YOLO

**DOI:** 10.1371/journal.pone.0320344

**Published:** 2025-04-18

**Authors:** Shiwei Yu, Feng Pan, Xiaoqiang Zhang, Linhua Zhou, Liang Zhang, Jikui Wang

**Affiliations:** CGN Digital Technology Co., Ltd., Shanghai, China; Zhejiang University, China

## Abstract

Aiming at the problems of low accuracy and large computation in the task of PCB defect detection. This paper proposes a lightweight PCB defect detection algorithm based on YOLO. To address the problem of large numbers of parameters and calculations, GhostNet are used in Backbone to keep the model lightweight. Second, the ordinary convolution of the neck network is improved by depthwise separable convolution, resulting in a reduction of redundant parameters within the neck network. Afterwards, the Swin-Transformer is integrated with the C3 module in the Neck to build the C3STR module, which aims to address the issue of cluttered background in defective images and the confusion caused by simple defect types. Finally, the PANet network structure is replaced with the bidirectional feature pyramid network (BIFPN) structure to enhance the fusion of multi-scale features in the network. The results indicated that when comparing our model with the original model, there was a 47.2% reduction in the model’s parameter count, a 48.5% reduction in GFLOPs, a 42.4% reduction in Weight, a 2.0% reduction in FPS, and a 2.4% rise in mAP. The model is better suited for use on low-arithmetic platforms as a result.

## 1. Introduction

With the development of the electronics industry, it has become an important component of modern manufacturing. Printed Circuit Boards (PCBs) are crucial electronic components that provide circuit connections and hardware support for devices. However, PCBs are typically made up of materials such as glass fibres, composite epoxy resins, and laminates. The manufacturing process is very complex and prone to errors, resulting in the presence of defects on the surface of PCBs [1]. Therefore, in order to ensure the safety and reliability of electronic products, it is imperative to conduct surface defect detection on PCBs before they leave the factory [2].

Traditional manual detection is easily influenced by external factors, which affects the quality and efficiency of the detection and introduces uncertainty [3,4]. Later on, due to the development of electronic devices, researchers started using image detection instead of manual detection [5]. Liu and Qu processed PCB images using a hybrid recognition method of mathematical morphology and pattern recognition and labelled PCB defect images for defect identification using an image aberration detection algorithm [6]. Khlong Luang and Pathum Thani proposed a PCB defect classification method using arithmetic and logical operations, Circular Hough Transform (CHT), Morphological Reconstruction (MR), and Connected Component Labelling (CCL) [7]. Mukesh Kumar et al. proposed a method for detecting defects in bare PCBs by combining image enhancement techniques with standard template generation particle analysis [8]. Although the accuracy of these methods reaches the detection needs, the detection speed is low, which is more limited in the actual detection. Currently, there has been rapid progress in the field of deep learning, with researchers successfully applying it to the task of defect detection in target objects [9]. Kuo et al proposed a graph convolutional network to detect components on printed circuit boards [10]. Liang and Gu et al proposed a multi-task learning model for locating and identifying waste materials simultaneously [11]. Nowakowski et al used CNN (a deep learning convolutional neural network) to identify and classify specific types of e-waste [12]. However, the application in PCB defect detection is limited by the computational power and time delay. The existing mainstream deep learning object detection algorithms can be mainly classified into two categories: one category is represented by the R-CNN series, which are two-stage algorithms [13]. This algorithm utilizes a region proposal network in the first stage to generate a large number of anchor boxes. The second stage involves performing classification and regression operations on these anchor boxes. Niu et al. proposed an improved PCB defect detection algorithm based on Faster RCNN [14]. They replaced ordinary convolutions with depthwise separable convolutions to reduce computational complexity. They also improved the feature pyramid to extract features at several depths, effectively combining low-level geometric details with semantics. Zeng et al. proposed an enhanced multi-scale feature fusion algorithm based on an asymmetric balanced feature pyramid network [15]. They utilized dilated convolutions to capture abundant contextual information and achieved effective PCB defect detection. Zhang et al. proposed a cost-sensitive residual convolutional neural network that effectively balances the different misclassification costs of sample imbalance and false defects in PCB detection [16]. Another category is represented by first-stage algorithms such as YOLO (You Only Look Once) and SSD (Single Shot MultiBox Detector) [17,18]. These algorithms directly utilize convolutional neural networks to extract target features and perform classification and regression on the targets. Li J et al. propose an improved algorithm based on YOLOv3, which uses a real PCB picture and a virtual PCB picture with synthesized data as a joint training dataset, which greatly increases the recognizability of training electronic components and provides the greatest possibility for data enhancement [19]. Lim JY et al. propose an enhanced deep learning network that addresses the difficulty in inferring tiny or varying defects on a PCB in real-time [20]. The proposed model is capable of performing accurate and reliable real-time PCB inspection with the aid of an automated alert capability. Chen W et al. propose a Transformer-YOLO network detection model to solve the problem of low accuracy and efficiency in printed circuit board (PCB) defect detection [21]. The network model better balances the detection accuracy, detection speed, and the volume. Jiang W et al. A joint multiscale PCB defect target detection and attention mechanism. The article built a feature fusion module to efficiently fuse low-level feature information with high-level feature information to produce a more complete feature map and improve the accuracy of fault recognition [22].

In order to address the above issues, this paper proposes a lightweight PCB surface defect detection model for low-computing power devices based on the YOLOv5 model.

## 2. Proposed Methods

The YOLO series algorithm is a single-step object detection algorithm based on convolutional neural networks, which significantly improves speed compared to two-step object detection algorithms. The network architecture of YOLOv5 consists of four components: input, backbone, neck, and prediction. The first part of the input includes the Mosaic data enhancement method and adaptive anchor box calculation. The second part of the system is Backbone, which consists of the Focus structure, CSP structure, and SPP structure, primarily used for feature extraction. The Focus structure deepens the feature dimension of the input image through slice operations. The CSP architecture divides the feature maps of the base layer into two parts and then merges them through a cross-stage hierarchical structure. The SPP module extracts feature information at different scales, increasing the receptive field and enriching the expressive power of the feature map. The third part Neck is the feature fusion part which includes the FPN+PAN structure. The FPN architecture efficiently conveys strong semantic features from top to bottom, aggregating parameters from different backbone layers to different detection layers, thereby enhancing the network’s ability to extract features. The fourth part Prediction includes Bounding box loss function calculation and NMS non-maxima suppression. The GIOU_Loss is used as the loss function for bounding box in Yolov5. During the post-processing stage of object detection, NMS is mostly used for filtering bounding boxes. Yolov5 employs a weighted NMS approach.

**Fig 1:**
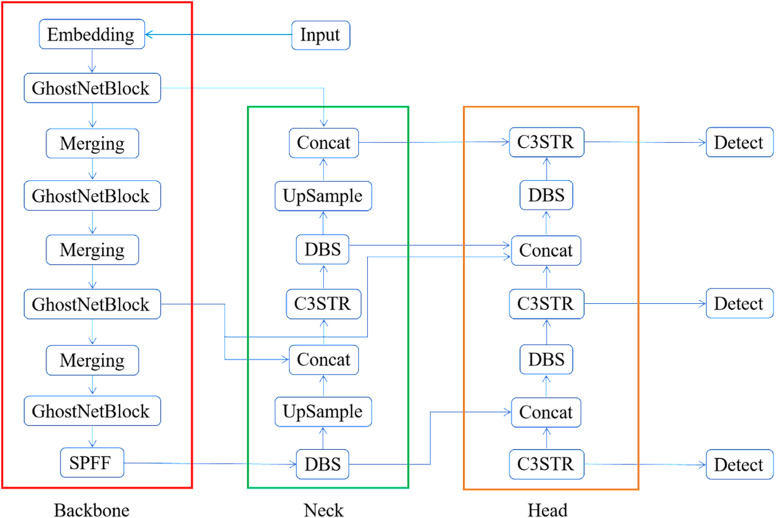
The network structure of our model.

This article proposes an enhanced model based on a single-stage detector, which improves classification accuracy and defect detection speed. Additionally, the computational complexity has been reduced. To address the problem of large numbers of parameters and calculations, GhostConv and GhostBottleneck are used in Backbone to keep the model lightweight. The improved YOLOv5 network is more suited for industrial applications in the real world due to its lighter network structure and lower hardware requirements. Second, slow model detection is caused by the YOLOv5 neck network’s CBS structure’s high memory utilization and a huge number of ordinary convolutional parameters. The number of parameters in the neck network can be efficiently decreased and the model detection speed increased by using DSConv to improve the ordinary convolution in the CBS structure. Afterwards, convolutional neural networks are prone to lose a large amount of high-level feature information after many convolution operations, resulting in a decrease in the detection ability of the network. Therefore, through the multi-head self-attention force Swin Trans-former module across the inter-window information interoperability characteristics, and the original C3 module fused with the composition of the new module C3STR, in order to better retain the global feature information at all scales. Solves the problem of confusion caused by background clutter and simple defect types in defect images. Finally, the PANet network structure is replaced with the bidirectional feature pyramid network (BIFPN) structure to enhance the fusion of multi-scale features in the network. During the network training process, because of the different sizes of different targets, it leads to the fact that the features of large targets can be retained as the convolution goes deeper during the convolution process, while the features of small targets may disappear. Therefore, it is necessary to fuse feature layers of different depths of the same target. Although PANet can effectively fuse different feature layers, it is still a simple addition of different features. However, due to the different sizes of the detected targets in different images, features with different resolution sizes are generated during training. They are still simply summed in PANet, which will result in unequal weighting of different sized features of the same type on the fused output. Large size features are incorporated more into the network while small size features contribute less. In order to enhance the detector’s ability to adapt to targets of different scales by integrating features from multiple scales. This helps to address the challenge of large differences in defect scales and poor detection capabilities for small defects. The network structure of our model is shown in Fig 1.

### 2.1. GhostNet-based backbone network improvement

MobileNet, ShuffleNet, EfficientNet, etc. are often used in the detection of surface defects in lightweight hot-rolled strips [23–25]. The use of DWConv deep convolution or GConv group convolution has been achieved to lighten the model detection task [26,27].

**Fig 2:**
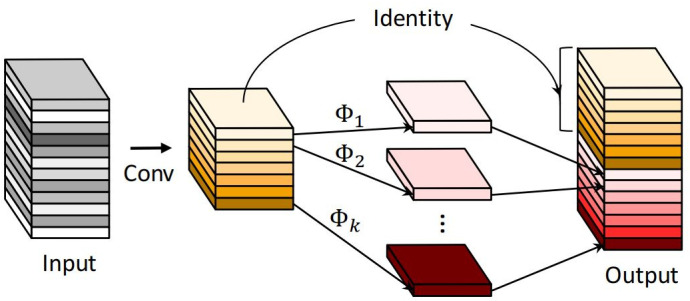
The structural layout of the Ghost module.

GhostNet is a lightweight convolutional network architecture proposed by Han K, et al [28]. The Ghost module is also known as Ghost Convolution. The fundamental concept is to improve the computational efficiency of the network by using ordinary linear variation to obtain redundant feature maps. Fig 2 depicts the structural layout of the Ghost module.

The specific process of GhostNet is


$$Y^{\prime }=X*f^{\prime }$$

$$\begin{array}{c} y_{ij}=\Phi_{ij}\left(y_{i}^{\prime }\right),i\in 1,2,\ldots ,m,j\in 1,2,\ldots ,s \end{array}$$

The first step is the convolution operation, X is the input feature map, $Y^{\prime }$ is the output m feature maps, and $f^{\prime }$ is the convolution kernel of size K×K. The second operation is the linear operation $\Phi_{ij}$ for each feature map $y_{i}^{\prime }$ in $Y^{\prime }$, and finally n=ms output feature maps $Y=[Y_{11},Y_{12},\ldots ,Y_{ms}]$ are obtained, and after calculation, it can be concluded that the ordinary convolution operation is about s times of Ghost module.

**Fig 3:**
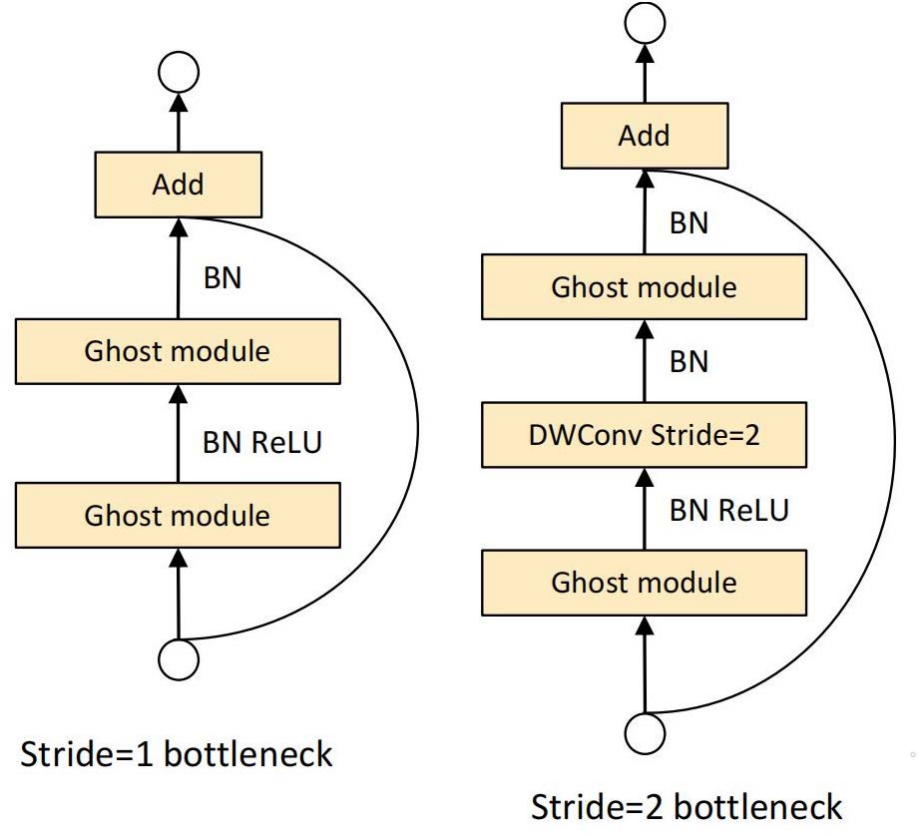
Ghost bottleneck. Left: Ghost bottleneck with stride = 1; right: Ghost bottleneck with stride = 2.

The GhostBottleneck module is the bottleneck layer in the GhostNet network architecture. It is composed of two Ghost modules that are stacked. By substituting the features generated by direct linear transformation for the ones provided by ordinary convolution, the model’s complexity is significantly reduced. The first Ghost module is followed by the Relu activation function, and the second Ghost module is followed only by the batch normalisation process. This structure not only reduces the model parameters and computation but also optimises the feature maps through the Ghost modules and improves the detection efficiency of the model. In addition, the two Ghost modules in Ghost Bottleneck can be connected by a deep convolution with stride =  2. Fig 3 depicts the structural layout of the GhostBottleneck module.

### 2.2. Improvement of neck networks based on depth-separable convolution

Slow model detection is caused by the YOLOv5 neck network’s CBS structure’s high memory utilization and a huge number of ordinary convolutional parameters. The ordinary convolution in the CBS structure is lightened and improved to decrease the number of neck network parameters and increase the speed of model detection. Depthwise Separable Convolution (DSConv) uses less resources and requires few memory [29]. The number of parameters in the neck network can be efficiently decreased and the model detection speed increased by using DSConv to improve the ordinary convolution in the CBS structure.

There are two stages in DSConv. Depthwise convolution is the initial step. This step uses a different convolution kernel to conduct convolution for each input channel. Pointwise convolution is the second step. This step uses convolution to modify the number of channels based on the pointwise convolution findings.

Assume $D_{F}$ is the input feature size, M is the number of input channels, $D_{H}$ is the output feature size, N is the number of output channels, and $D_{K}$ is the convolution kernel size. The computation of ordinary convolution $C_{1}$ is:


$$\mathrm{\backslash [}C_{1}=M\times D_{K}\times D_{K}\times N\times D_{F}\times D_{F}\mathrm{\backslash ]}$$

The computation $C_{2}$ of DSConv is:


$$\mathrm{\backslash [}C_{2}=M\times D_{K}\times D_{K}\times D_{F}\times D_{F}+M\times N\times D_{F}\times D_{F}\mathrm{\backslash ]}$$

The ratio of DSConv to ordinary convolutional computation is:


$$\mathrm{\backslash [}\frac{C_{2}}{C_{1}}=\frac{M\times D_{K}\times D_{K}\times D_{F}\times D_{F}+M\times N\times D_{F}\times D_{F}}{M\times D_{K}\times D_{K}\times N\times D_{F}\times D_{F}}\mathrm{\backslash ]}$$

From the above equation, it can be seen that the computational volume of DSConv is much less than that of normal convolution.

**Fig 4:**
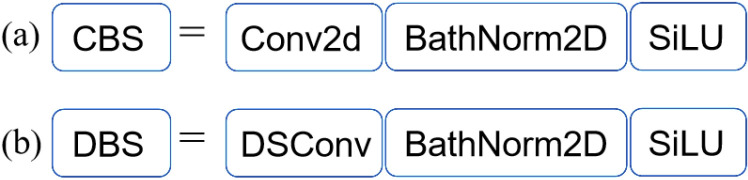
The CBS structure improvement based on DSConv.

Fig 4 displays the improved CBS model’s structure based on DSConv. By adding DSConv to the YOLOv5 neck network, the CBS structure’s regular convolution is reconstructed into depthwise and pointwise convolutions. This structure is defined as DBS. Model detection speed can be increased by decreasing the amount of parameters and memory utilisation of the YOLOv5 neck network.

### 2.3. C3STR module incorporating Swin-Transformer

**Fig 5:**
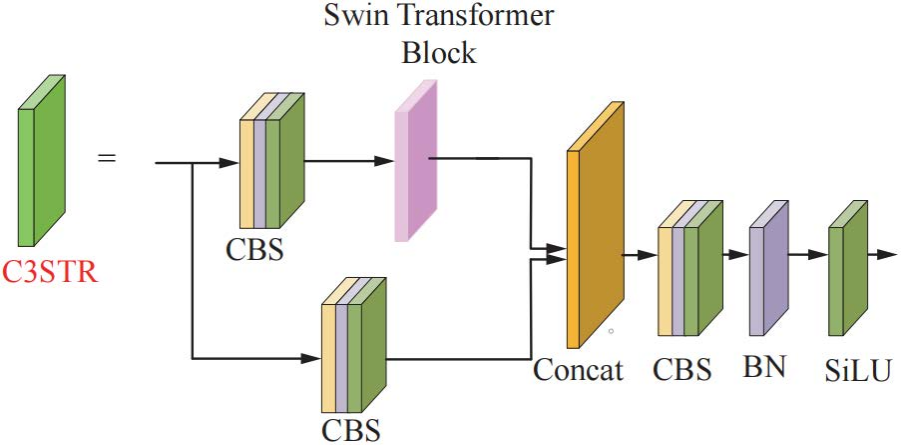
The improved C3STR structure.

Convolutional neural networks are prone to lose a large amount of high-level feature information after many convolution operations, resulting in a decrease in the detection ability of the network. Therefore, through the multi-head self-attention force Swin Trans-former [30] module across the inter-window information interoperability characteristics, and the original C3 module fused with the composition of the new module C3STR, in order to better retain the global feature information at all scales. The improved C3STR structure is shown in Fig 5.

The Swin-Transformer module is composed of paired Window Multi-head Self-Attention (W-MSA), Shifted Window Multi-head Self-Attention (SW-MSA) and Multi-Layer Perceptron (MLP) blocks. The sublayers between each module are connected using residual edges. The W-MSA module first divides the windows in different regions and then completes the computation of self-attention separately. This can greatly reduce the computational complexity and improve the training speed of the network. SW-MSA module adds the offset mechanism, i.e., sliding window operation mechanism, based on W-SMA module. SW-MSA first re-slices the window partitions between the continuous self-consciousness layers and obtains the new window layout again by cyclic shifting of the sliding method. Then use the cross-window connection technique to recalculate the attention weights inside each newly generated window. In order to achieve the feature unit to exchange feature information between different windows, capture more different contextual information, to improve the ability to obtain the global information of the network. By limiting the computation to windows, these two sub-modules considerably lower the computational complexity when compared to the Transformer self-attention MSA sub-module.

### 2.4. Using the BiFPN network

**Fig 6:**
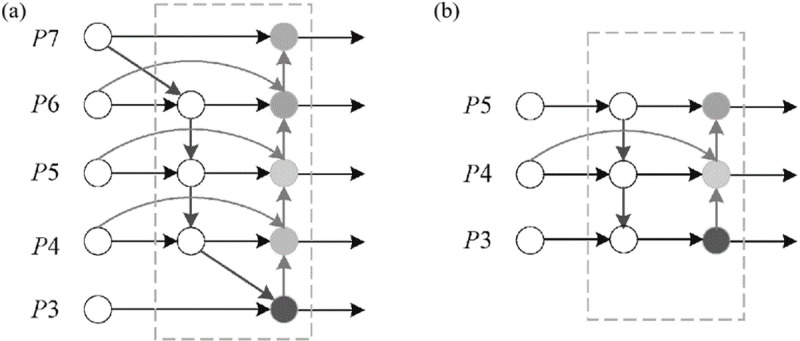
(a) The network structure of BiFPN; (b) The BiFPN network structure used in this paper.

During the process of convolution, the fine details of low-level features are often lost in the downsampling process [31]. To obtain feature maps with richer semantic information, replace PANet in the Neck with BiFPN. The use of a bidirectional network connection method based on the weighted bidirectional feature pyramid structure (BiFPN) improves the effectiveness of surface defect detection on PCB boards. The main idea of BiFPN is the bidirectional cross-scale connections and weighted feature fusion [32]. The network structure of BiFPN and the BiFPN network structure used in this paper is shown in Fig 6.

$P_{6}^{td}$is the intermediate feature of P6 layer and $P_{6}^{in}$ and $P_{6}^{out}$ are the input and output features. The band-weighted feature fusion formula for BiFPN is:


$$\mathrm{\backslash [}P_{6}^{td}=Conv\left(\frac{\omega_{1}\cdot P_{6}^{in}+\omega_{2}\cdot Resize\left(P_{7}^{in}\right)}{\omega_{1}+\omega_{2}+\varepsilon }\right)\mathrm{\backslash ]}$$

$$\mathrm{\backslash [}P_{6}^{td}=Conv\left(\frac{\omega_{1}^{\prime }\cdot P_{6}^{in}+\omega_{2}^{\prime }\cdot P_{6}^{td}+\omega_{3}^{\prime }\cdot Resize\left(P_{5}^{out}\right)}{\omega_{1}^{\prime }+\omega_{2}^{\prime }+\omega_{3}^{\prime }+\varepsilon }\right)\mathrm{\backslash ]}$$

Where: Conv is the corresponding convolution operation; Resize is the up-sampling or down-sampling operation; ω is the corresponding weight of each layer, which is used to distinguish the importance of different features in the process of feature fusion; and ε is a very small non-zero number.

BiFPN networks possess the ability to perform feature fusion at several levels across various scales, while also exhibiting bidirectional connectivity. The model eliminates the P3 and P7 layer feature fusion nodes that have minimal contribution to the network in order to decrease the computational workload. Additionally, it incorporates an edge to connect the input deterioration outputs. Acquire profound semantic data and preserve additional geographical details without incurring additional expenses. Utilizing the fusion of shallow feature maps enhances the precision of detecting small targets. This leads to a decrease in the expenses associated with processing and storage, while simultaneously enhancing the precision of detection.

## 3. Experiments

### 3.1. Datasets

**Fig 7:**
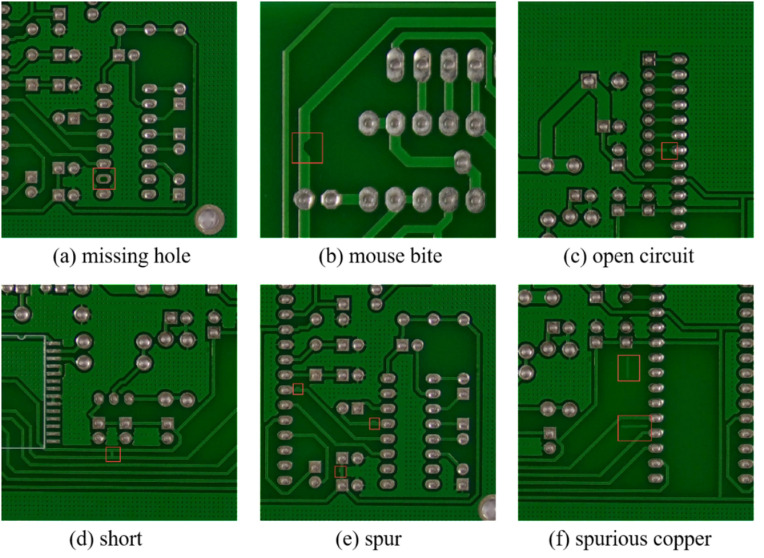
The defect images in the PCB defect dataset.

PKU-Market-PCB is a publicly available dataset from Peking University’s Open Laboratory for Intelligent Robotics, and it is a publicly synthesized PCB defect dataset [33]. The dataset contains 693 images of PCB defects that were cropped to produce 10,668 images. The dataset consists of 10,668 images, each of which contains one of six types of defects: missing hole (Mh), mouse bite (Mb), open circuit (Oc), short (Sh), spur (Sp), and spurious copper (Sc). Table 1 shows the number of images for each defect type. Defects in the PCB images were labelled using the LabelImg tool and stored in the Pascal VOC dataset format. The dataset was then divided into a training set and a test set in a ratio of 8:2. Fig 7 shows the defect images in the PCB defect dataset.

**Table 1:** Experimental environment configuration.

experimental environment	configuration
CPU	AMD R7 4800H CPU
GPU	NVIDIA GeForce RTX2060 GPU 6 GB
random access memory	16 GB RAM
programming language	Python 3.8.13
deep Learning Framework	PyTorch 1.12.1
CUDA	CUDA 11.7

### 3.2. Training details

The specific configuration of the experimental environment is shown in Table 1.

The parameter settings for this experiment are shown in Table 2.

**Table 2:** Experimental parameter settings.

parameter name	parameter value
training epoch	300
image size	640 × 640
batch_size	16
learning rate	0.01
momentum	0.937
weight decay	0.0005

Due to the small dataset, the training process employs a transfer learning method, where pre-trained weight parameters are loaded on the PCB surface defect dataset. At the beginning of the training, there is a large distance between all the parameters obtained randomly and the final training result. Training with a very large learning rate will result in unstable values. So a small learning rate is used at the beginning to get closer to the approximate location of the solution space, and at this stage the learning rate is increased to the initially set value. Throughout the initial three training Epochs, the model’s learning rate gradually increases. Upon the completion of three Epochs, the learning rate will be modified to 0.01.

## 4. Results and discussions

### 4.1. Experimental results and ablation study

Table 3 shows the specific parameters of the model and the results of the ablation experiments. Our model parameters decreased by 47.2%, GFLOPs decreased by 48.5%, Weight decreased by 42.4%, mAP increased by 2.4%, and FPS decreased by 2.0% when compared to the YOLOv5s model. The introduction of GhostNet achieves a lightweight model. PConv uses ordinary convolution for spatial feature extraction on only some of the input channels. Keeping the rest of the channels unchanged at the same time ensures that the inputs and outputs have the same number of channels. The computational complexity of the model is effectively reduced while preserving the spatial information. When the model outputs feature maps, there are many output features that are very similar. These similar feature maps can basically be obtained by simple linear transformations, without the need for complex non-linear transformations. One of the feature maps can be obtained by cheaply transforming another feature map, and one of the feature maps can be considered the ‘Ghost’ of the other. Therefore, since some of the feature maps are not obtained by convolution operation, a cheaper operation is used to generate the ‘Ghost’ feature maps. Therefore, the missing part of the information will reduce the quality of feature extraction when the PCB background is cluttered, resulting in a slight decrease of mAP from 94.8% to 94.2%. Lightweight improvement of the ordinary convolution in the CBS structure by using DSConv. It effectively reduces the number of parameters in the neck network and improves the model detection speed. By fusing Swin-Transformer with the C3 module on the neck. The parameters and GFLOPs had a small rise, while the modeled mAP showed a significant improvement of 3.3%. It effectively enhances the model’s ability to deal with the cluttered background of defect images and the easy confusion of defect types. Replacing the PANet in the neck network with a BiFPN resulted in a 1.1% increase in the model’s mAP. This is because BiFPN networks can perform multi-level feature fusion across scales while having bi-directional connectivity. Gain deep semantic information and retain more positional information. The shallow feature maps are fused to improve the detection accuracy of small targets such as PCB surface defects. In contrast to PANet, BiFPN references Attention to increasing the weights of the fused features of different sizes. It dynamically learns to adjust the contribution of each scale so that the network can better integrate features of different sizes as they become available. At the same time, it adds residual connections to enhance the feature representation.

**Table 3:** Specific parameters of each model and ablation experiments.

Method	Parameters(M)	GFLOPs	Weight(M)	mAP(%)	FPS
YOLOv5s(baseline)	7.08	16.5	14.4	94.8	5.1
+GhostNet	4.17	9.3	8.7	94.7	5.0
+DBS	6.39	15.6	13.8	94.2	5.7
+ C3STR	7.29	17.0	15.0	97.9	4.6
+BiFPN	7.00	16.4	15.1	95.8	4.9
+GhostNet+DBS	4.11	8.5	7.7	94.2	5.4
+C3STR+BiFPN	7.20	16.7	15.1	98.1	4.8
OURS	3.74	8.5	8.3	97.1	5.0

**Fig 8:**
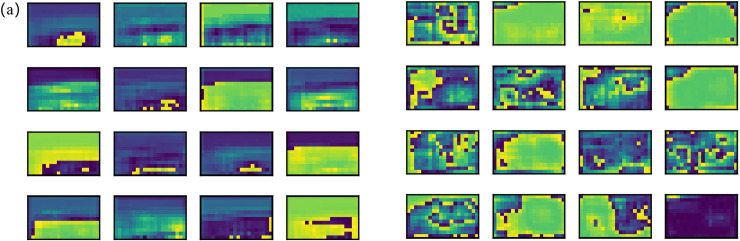
(a) Feature map before adding C3STR module; (b)Feature map after adding C3STR module.

In order to show the effect of C3STR directly, Fig 8 shows the visual comparison of the feature maps before and after adding the C3STR module. It can be seen from the figure that the addition of C3STR makes the model acquire more detailed information about the target and the target features are more obvious. C3STR enables the algorithm to have a larger sensory field while fully retaining more strong semantic information in the feature map. This is more conducive to recovering the feature information at the defects in order to obtain high-quality feature maps of PCB surface defects, which provide rich feature map information for the lower-level detection tasks. It achieves a more accurate localisation of the defect target, thus improving the detection accuracy of the network.

Our model’s mAP compared to the six defects of YOLOv5s is shown in Table 4. The mAP of Mh, Mb, Oc, Sh, Sp, and Sc increased by 1.9%, 3.4%, 1.4%, 1.9%, 2.5%, and 3.6%, respectively. Mb, Sp, and Sc show significant improvement, whereas the improvement in Mh, Oc, and Sh is not evident. The former features are less obvious and belong to small targets, and the model improves the detection of small targets, so the lift is higher. The latter features are more obvious, so the lift is not high.

**Table 4:** The effect of Faster-SWDB—YOLO on 6 types of defects detection.

Method	Mh	Mb	Oc	Sh	Sp	Sc
YOLOv5s	96.5	93.9	96.1	96.4	94.5	91.4
OURS	98.3	97.1	97.4	98.2	96.9	94.7

**Fig 9:**
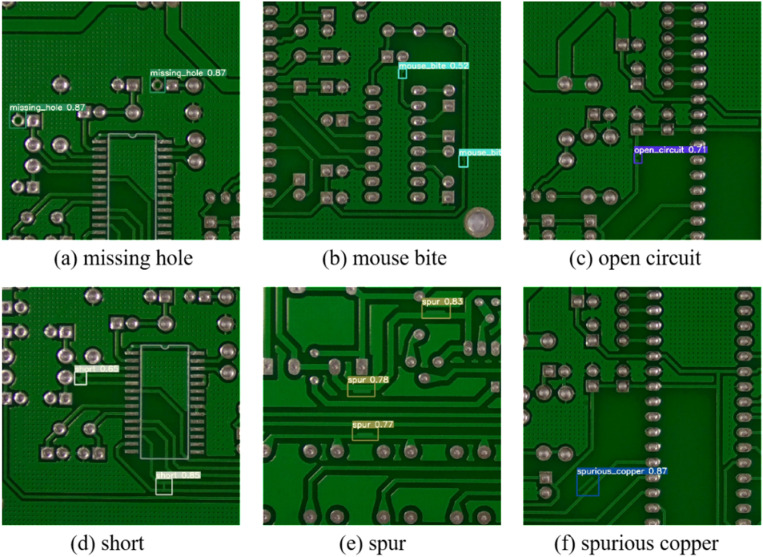
The detection results of our model.

Based on the above analysis, the inclusion of GhostNet and DBS has effectively reduced the parameters, GFLOPs, and weight of the model. The introduction of Swin-Transformer and BiFPN significantly improves the model’s mAP. Fig 9 depict some detection results of our model.

### 4.2. Comparison of different algorithms

In this paper, we choose SSD, Faster R-CNN, Retina-Net, YOLOv3, YOLOv4, YOLOv7, and YOLOv8 to compare the performance with our model. Table 5 demonstrates that our model’s mAP surpasses that of SSD, Faster-RCNN, Retina-Net, YOLOv3, YOLOv4, and YOLOv7 by a significant margin. The SSD uses a multi-layer feature fusion approach. The feature extraction part of SSD uses VGG as the main part. Due to the low network complexity of VGG, it is difficult to extract the key target feature content in the PCB. For small targets such as PCB defects, their features cannot be fully extracted, resulting in a mAP that is 25.8% lower than our model when Parameters, GFLOPs, and FPS are much higher. Faster R-CNN is a two-stage detection algorithm. Candidate frames are first generated and then classification and regression operations are performed to get the location and category of the target. The tracing frame mechanism of Faster R-CNN achieves comprehensive recognition of targets in the inspected image by traversing the feature map. However, due to the complex background of PCB boards, the difference between defective targets and normal targets is small. When the tracing frame starts traversing, the selected targets may contain normal regions, resulting in a larger impact of the detection model on the category accuracy rate. Meanwhile, the algorithm is computationally intensive, and the mAP and FPS are 18.0% and 70.0% lower than our model, respectively. The detection speed is difficult to meet the requirement of real-time detection. The YOLOv3 is poor at capturing small targets. The detection accuracy of YOLOv4 is 12.7% lower than our model, while the detection speed is also low, making it difficult to deploy. YOLOv7 adopts a new feature extraction structure, ELAN, and designs a deeper network with an accuracy of 89.6%. However, the deeper network often tends to lead to the loss of small targets and edge information, which is not conducive to PCB defect detection. Additionally, the mAP of our model is slightly higher than YOLOv8 by 2.5%. This is due to the fact that most of the current methods for detecting PCB surface defects are only good for specific defect categories and lack good applicability to multiple categories of defects. We have made corresponding improvements to address specific needs. Moreover, the parameters and GFLOPs of the model is much higher than other models due to the introduction of GhostNet and DBS. This indicates that the model is capable of meeting the detection requirements of low computational power platforms.

**Table 5:** Performance of different algorithms.

Types	Mh	Mb	Oc	Sh	Sp	Sc	Parameters	GFLOPs	mAP	FPS
SSD	85.3	70.6	71.7	82	67.1	73.2	23.6	188.9	72.0	2.5
Faster-RCNN	91.7	78.2	80.6	84.8	74.8	85.1	7.6	169.7	79.6	1.5
Retina-Net	90.7	90	8735	90.5	88.3	85.8	36.7	104.2	85.9	3.5
YOLOv3	88.3	84.8	87.0	89.1	84.0	83.2	58.7	154.6	86.1	3.4
YOLOv4	86	82.8	85.3	91.4	86	94.8	44.3	114.1	84.8	2.7
YOLOv5	96.5	93.9	96.1	96.4	94.5	91.4	7.1	16.5	94.8	5.1
YOLOv7	99.5	91.8	93.1	93.6	83.9	93.1	34.8	103.2	89.6	2.9
YOLOv8	99.5	98.2	95.8	98.7	94.6	98.8	11.1	28.4	94.7	4.5
OURS	98.3	97.1	97.4	98.2	96.9	94.7	3.74	8.5	97.1	5.0

## 5. Conclusions

In this paper, a lightweight PCB defect detection algorithm based on YOLO is proposed. To address the problem of large numbers of parameters and calculations, GhostNet are used in Backbone to keep the model lightweight. Second, the ordinary convolution of the neck network is improved by depthwise separable convolution, resulting in a reduction of redundant parameters within the neck network. Afterwards, the Swin-Transformer is integrated with the C3 module in the Neck to build the C3STR module, which aims to address the issue of cluttered background in defective images and the confusion caused by simple defect types. Finally, the PANet network structure is replaced with the bidirectional feature pyramid network (BIFPN) structure to enhance the fusion of multi-scale features in the network. The results indicated that when comparing our model with the original model, there was a 47.2% reduction in the model’s parameter count, a 48.5% reduction in GFLOPs, a 42.4% reduction in Weight, a 2.0% reduction in FPS, and a 2.4% rise in mAP. The model is better suited for use on low-arithmetic platforms as a result. The experimental results show that the improved lightweight network obtains better detection results. However, there is still potential for further improvements in terms of detection speed and efficiency. In the next study, we will introduce richer datasets to strengthen their generalisation ability.
